# Intrauterine growth restriction combined with a maternal high‐fat diet increases hepatic cholesterol and low‐density lipoprotein receptor activity in rats

**DOI:** 10.14814/phy2.12862

**Published:** 2016-07-12

**Authors:** Erin K. Zinkhan, Jennifer M. Zalla, Jeanette R. Carpenter, Baifeng Yu, Xing Yu, Gary Chan, Lisa Joss‐Moore, Robert H. Lane

**Affiliations:** ^1^ Division of NeonatologyDepartment of PediatricsUniversity of UtahSalt Lake CityUtah; ^2^ Division of Maternal Fetal MedicineDepartment of Obstetrics and GynecologyUniversity of UtahSalt Lake CityUtah; ^3^ Department of PediatricsMedical College of WisconsinMilwaukeeWisconsin

**Keywords:** Cholesterol, intrauterine growth restriction, Ldlr, maternal high‐fat diet

## Abstract

Intrauterine growth restriction (IUGR) and maternal consumption of a high‐saturated‐fat diet (HFD) increase the risk of hypercholesterolemia, a leading cause of morbidity and mortality. Many pregnant women eat a HFD, thus exposing the fetus to a HFD in utero. The cumulative effect of in utero exposure to IUGR and a HFD on offspring cholesterol levels remains unknown. Furthermore, little is known about the mechanism through which IUGR and maternal HFD consumption increase cholesterol. We hypothesize that IUGR combined with a maternal HFD would increase offspring serum and hepatic cholesterol accumulation via alteration in levels of key proteins involved in cholesterol metabolism. To test our hypothesis we used a rat model of surgically induced IUGR and fed the dams a regular diet or a HFD. HFD‐fed dams consumed the same kilocalories as regular diet‐fed dams, with no difference between surgical intervention groups. In the offspring, IUGR combined with a maternal HFD increased hepatic cholesterol levels, low‐density lipoprotein (LDL) receptor protein levels, and Ldlr activity in female rat offspring at birth and both sexes at postnatal day 14 relative to non‐IUGR offspring both from regular diet‐ and HFD‐fed dams. These findings suggest that IUGR combined with a maternal HFD increases hepatic cholesterol accumulation via increased LDL cholesterol uptake into the liver with resulting persistent increases in hepatic cholesterol accumulation.

## Introduction

Fetal intrauterine growth restriction (IUGR) occurs in approximately 7–10% of the population and increases the risk of developing hypercholesterolemia in adulthood (Forsdahl 1978; Barker et al. 1989, 1993; King 2006; Demicheva and Crispi 2014). The risk of the IUGR individual developing adult‐onset hypercholesterolemia is potentiated by maternal consumption of a high‐saturated‐fat diet (HFD) (Napoli et al. 1997, 1999; The Hertfordshire Cohort Study, 2006). Many IUGR infants are exposed to a maternal HFD in utero. The average American consumes approximately 23–33 g of saturated fat and up to 400 mg cholesterol daily, both of which exceed the recommended fat and cholesterol intake of approximately 16 g saturated fat and 200 mg cholesterol (Wright et al. 2003; Reynolds et al. 2013). Despite the broad public health implications through which a maternal HFD exacerbates the IUGR predisposition toward adult‐onset hypercholesterolemia, the mechanisms through which the fetal environment impacts cholesterol metabolism remain unclear (the Hertfordshire Cohort Study, 2006; Zinkhan et al. 2014).

**Figure 1:**
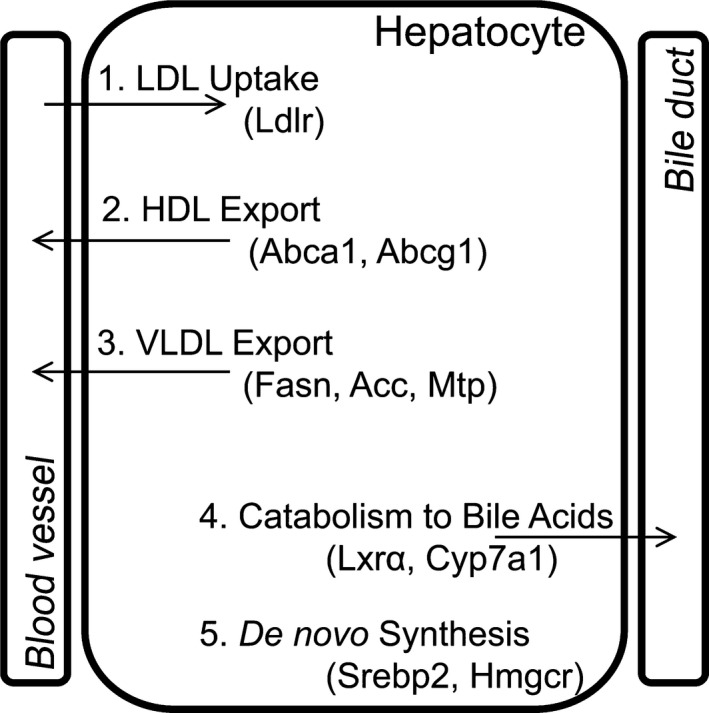
Schematic of major cholesterol metabolism pathways in the liver. The first pathway is LDL uptake from the blood via Ldlr. The second pathway is HDL export to the blood via Abcg1 and Abca1. The third pathway is VLDL export to the blood via Fasn, Acc, and Mtp. The fourth pathway is cholesterol catabolism to bile acids via Lxr*α* and Cyp7a1. The fifth pathway is de novo synthesis of cholesterol via Srebp 2 and Hmgcr. LDL, low‐density lipoprotein; HDL, high‐density lipoprotein; VLDL, very low‐density lipoprotein.

The liver contributes significantly to the regulation of serum cholesterol levels. The liver regulates cholesterol levels through five major pathways, and alteration of these pathways can cause cholesterol accumulation in the liver (Fig. 1). The first major pathway through which the liver regulates cholesterol is low‐density lipoprotein (LDL) uptake from the blood via the LDL receptor (Ldlr). The second pathway is high‐density lipoprotein (HDL) export to the blood via ATP‐binding cassette transporter g1 (Abcg1) and Abca1. The third pathway is very low‐density lipoprotein (VLDL) export to the blood via fatty acid synthase (Fasn), acetyl‐coA carboxylase (Acc), and microsomal transferase protein (Mtp). The fourth pathway is cholesterol catabolism to bile acids via key proteins liver X receptor *α* (Lxr*α*) and cholesterol 7 alpha‐hydroxylase (Cyp7a1). Finally, the fifth pathway is de novo synthesis of cholesterol via sterol‐responsive element‐binding protein (Srebp) 2 and 3‐hydroxy‐3‐methylglutaryl‐CoA reductase (Hmgcr). These five pathways work in concert to regulate serum and hepatic cholesterol levels.

To begin to study the mechanism through which IUGR and a maternal HFD programs increased cholesterol, we developed a physiologically relevant, unique model of IUGR combined with maternal HFD feeding from 5 weeks prior to conception through lactation. We hypothesized that compared to IUGR alone and a maternal HFD alone, IUGR combined with a maternal HFD would increase serum and hepatic cholesterol in association with altered protein levels of key regulators of cholesterol metabolism in both fetal and juvenile rats.

## Methods

### Animal husbandry and study design

The University of Utah Animal Care Committee approved all animal interventions and procedures. Fifty‐day‐old male and nonpregnant female Sprague Dawley rats were obtained from Charles River Laboratories, Inc. (Wilmington, MA). Rats were housed individually and exposed to 12‐h light–dark cycles. Male rats were kept on a regular rat chow (Reg, Harlan Teklad, TD.8640, Madison, WI). Female rats were placed on one of two diets, a regular rat chow (TD.8640) or a high‐fat diet rat chow (Harlan Teklad, TD.110526). The regular chow contained 54% kcal carbohydrate, 17% kcal from fat, and 29% kcal from protein. The fat source in the regular rat chow was soybean oil at 60 g/kg food and contains 0.03% wt/wt cholesterol. The HFD contained 40% kcal from carbohydrate, 44% kcal from fat, and 16% kcal from protein. The fat source in the HFD was soybean oil at 10 g/kg and milk fat, for a total of 65% saturated fat. The HFD contained 1% wt/wt cholesterol and 0.5% cholic acid. Protein content in the HFD was lower than the Reg diet. This protein content was chosen to mimic protein consumption in the United States at present (Wright et al. 2003) and is sufficient for normal mammalian growth (FAO, WHO, 1973). Furthermore, this protein content is significantly higher than protein content used in low‐protein dietary studies (Boujendar et al. 2002).

After 5 weeks on either the regular diet or HFD, males were placed in the same cage as females overnight. Pregnancy day 0.5 was determined by the presence of sperm on a vaginal swab. Bilateral uterine artery ligation was performed on embryonic day 19.5 (E19.5) of a 21.5‐day gestation as described previously (Fu et al. 2006). Anesthesia alone was used as a control as previously sham surgery also induces growth restriction in the offspring (Ogata et al. 1986). This study design resulted in four maternal groups: regular diet‐fed dams given anesthesia alone (Anesthesia+Reg), regular diet‐fed dams that underwent IUGR surgery (Surgery+Reg), HFD‐fed dams given anesthesia alone (Anesthesia+HFD), and HFD‐fed dams that underwent IUGR surgery (Surgery+HFD). In all, 24–28 dams per diet and surgical intervention were used to generate offspring for these studies, with a minimum of six dams per time point. Offspring were harvested at postnatal day (PND) 0 (fetal rat) or PND 14 (juvenile rat). Our study design resulted in four offspring groups per sex from PND 0 through 14: maternal regular diet‐fed control rats (Con+Reg), maternal regular diet‐fed IUGR rats (IUGR+Reg), maternal HFD‐fed control rats (Con+HFD), and maternal HFD‐fed IUGR rats (IUGR+HFD).

Rats for analysis at PND 0 were delivered via cesarean section at embryonic day 21.5 (E21.5) after a 6‐h maternal fast. At the time of harvest, pregnant dams were anesthetized with 8 mg/kg xylazine and 40 mg/kg ketamine prior to cesarean section of fetal rats. Only one male and one female fetal rat offspring per litter were used in these studies, for a total of six males and six females per group. The remainder of pregnant rats was allowed to deliver naturally and litters were culled to six at birth for rearing consistency. All rats were weighed weekly to follow growth. Rat food was weighed weekly to measure fat and cholesterol intake. Maternal diets were continued through PND 14. Offspring at PND 14 were separated from the dam, kept warm in a cage with other PND 14 rats, fasted for 4–6 h prior to harvest, and anesthetized with 8 mg/kg xylazine and 40 mg/kg ketamine prior to decapitation. Only one male and one female juvenile rat offspring per litter were used in these studies, for a total of six males and six females per group.

### Maternal milk analysis

The fat and cholesterol concentration in dam milk may impact cholesterol levels in the PND 14 offspring. On postdelivery day 14, dams were separated from their offspring for 2–4 h prior to milking. Dams had access to food and water ad libitum while separated from the offspring. One international unit of oxytocin was administered subcutaneously over the dam's flank. 5 min later, the dam was anesthetized with an intraperitoneal injection of 40 mg ketamine and 8 mg xylazine. Milk was hand expressed until the milk gland was empty and the milk was placed immediately on ice. Approximately 400–500 *μ*L of milk were obtained from each dam regardless of diet or surgery status. Milk was stored at −80°C until analysis. Dams were killed after milking.

Rat milk lipids were separated by centrifugation in a capillary tube, and the creamatocrit was expressed as percent of total milk volume comprised of lipids. Rat milk cholesterol was measured using a colorimetric kit (Cholesterol/Cholesteryl Ester Quantitation Colorimetric Kit II, Biovision, Biovision Research Products, Mountain View, CA) per the manufacturers’ instructions. Milk cholesterol was calculated as milligrams per deciliter and compared to reference data.

### Serum cholesterol analysis

Serum was collected at offspring necropsy in serum separator tubes (BD Vacutainer, BD, Franklin Lakes, NJ). Serum from pregnant dams was collected at the time of cesarean section. Serum aliquots were frozen at −80°C. Serum lipid panels for dams were analyzed at ARUP laboratories within 1 week of collection (ARUP lab number 0020421). Fetal and PND 14 rat serum yielded insufficient quantities for analysis at ARUP, therefore, offspring rat serum cholesterol was measured with a colorimetric kit (BioVision HDL‐C and LDL‐C/VLDL‐C Cholesterol Quantification Kit, Biovision Research Products) according to the manufacturers’ protocol.

### Hepatic cholesterol quantification

Flash‐frozen hepatic lipids were isolated based on the method developed by Folch et al. (1957). Cholesterol was quantified using a colorimetric kit (BioVision HDL‐C and LDL‐C/VLDL‐C Cholesterol Quantification Kit) according to the manufacturers’ protocol. Data were calculated as mg cholesterol/100 g liver weight and compared to reference data.

### Hepatic protein quantification

Protein from flash‐frozen livers was isolated using whole‐cell lysates in buffer (150 mmol/L NaCl, 50 mmol/L Tris pH 7.4, 1 mmol/L EDTA, 0.25% Na‐deoxycholate, 1% Igepal CA‐630). Protein concentrations were determined bicinchoninic acid assay with bovine serum albumin as a standard curve. A total of 50 *μ*g protein was run on a 10% SDS‐PAGE gel (Bio‐Rad, Hercules, CA). The protein was transferred to a PVDF membrane and blocked with 5% milk. Hprt1 (15059‐1‐AP, Proteintech, Chicago, IL) was used as a loading control, as Hprt1 did not differ by intrauterine conditions or diet. Antibodies used were Ldlr (3839‐100; BioVision, Milpitas, CA), Abca1 (NB400‐105; Novus Biologicals, Littleton, CO), Abcg1 (sc‐11150; SantaCruz Biotechnology), Acc (3662; Cell Signaling Technology, Danvers, MA), Fasn (610962, BD Transduction Laboratories, Santa Cruz, CA), Mtp (612022, BD Transduction Laboratories), Lxr*α* (LS‐C172075, lot 47132, LifeSpan BioSciences, Inc.), Cyp7a1 (sc‐25536, Santa Cruz Biotechnology, Inc.), Srebp2 (ab28482; Abcam, Cambridge, MA), and Hmgcr (NBP1‐50713, Novus Biologicals). Western lightning‐enhanced chemiluminescence (ECL) (PerkinElmer Life Sciences; Waltham, MA, USA) was used to detect protein using goat anti‐rabbit or anti‐mouse (for Fasn and Mtp) horseradish peroxidase‐conjugated secondary antibody from Cell Signaling Technology. A Kodak Image Station 2000ER (Eastman Kodak/SIS, Rochester, NY) was used to image and quantify protein.

### Quantification of hepatic Ldlr activity

Six pregnant dams per time point, diet, and surgical intervention were generated for the determination of hepatic Ldlr activity, which generated six offspring per sex, diet, and surgical intervention at each time point. These rats were not used in other experiments described in this manuscript.

Hepatic Ldlr activity was quantified in isolated primary hepatocytes using a fluorometric kit (BioVision LDL Uptake Assay Kit) according to the manufacturers’ protocol. In brief, fresh primary hepatocytes from 20 *μ*g liver were isolated from fetal and PND 14 rats using a 1‐h collagenase digestion followed by washing in PBS and plating on a 96‐well plate. After 4 h of starving the cells, fluorescently labeled LDL was added to the primary hepatocytes for a 2‐h incubation period. Fluorescence was measured using a spectrophotometer. After fluorescence measurement, cells in each well were lysed and protein quantified using a bicinchoninic acid assay with bovine serum albumin as a standard curve. Data were calculated as *μ*g LDL uptake/mg total protein/hour and compared to standard curve LDL uptake reference data.

### Statistics

Data were expressed as mean ± standard error of the mean (SE). Nonparametric statistics (ANOVA with Fisher's protected least‐significant difference) was used for data analysis. The statistic package StatView (SAS Institute Inc., Cary, NC) was used for analyses. A *P *≤* *0.05 was considered to be statistically significant.

## Results

### Effect of diet and surgery on pregnancy, maternal lipids, and milk composition

Nonpregnant females had the same body weight prior to pregnancy regardless of diet (Table 1). At the time of term gestation, pregnant dams on HFD weighed more than pregnant females on regular diet, despite consuming the same kilocalories and having the same litter size. HFD‐fed dams consumed significantly more fat and cholesterol than dams on the regular diet, with no difference between dams that underwent IUGR surgery and dams that received anesthesia alone.

**Table 1:** Maternal pregnancy characteristics and food intake

	Anesthesia+Reg	Anesthesia+HFD	Surgery+Reg	Surgery+HFD
Pre‐pregnancy weight (g)	269 ± 22	287 ± 22	309 ± 28	290 ± 19
Weight at term (g)	385 ± 15	422 ± 28^a^	398 ± 46	392 ± 25
Pregnancy weight gain (g)	115 ± 26	134 ± 9^a^	90 ± 39	102 ± 22
Number of pups per litter	13 ± 2	14 ± 4	13 ± 3	12 ± 2
Food intake (kcal/day)	65 ± 5	65 ± 3	63 ± 5	59 ± 5
Fat intake (kcal/day)	11 ± 1	29 ± 1^a^	11 ± 1	26 ± 2^a^
Cholesterol intake (mg/day)	1 ± 0	144 ± 7^a^	1 ± 0	131 ± 11^a^

HFD, high‐saturated‐fat diet; *N* = 10 rat dams per group. Data are shown as mean ± SEM.

a *P *≤* *0.05 compared to Anesthesia+Reg.

At the time of cesarean delivery, HFD dams had markedly increased fasting serum total cholesterol and non‐HDL cholesterol compared to regular diet‐fed dams (Table 2). Surgery+HFD dams had decreased total and non‐HDL cholesterol compared to Anesthesia+HFD dams. Compared to Anesthesia+Reg dams, Anesthesia+HFD dams had an increased ratio of non‐HDL to HDL cholesterol. Surgery+HFD dams had a decreased ratio of non‐HDL to HDL cholesterol compared to Anesthesia+HFD dams.

**Table 2:** Maternal fasting serum lipid profile

	Anesthesia+Reg	Anesthesia+HFD	Surgery+Reg	Surgery+HFD
Total cholesterol (mg/dL)	79 ± 5	684 ± 105^a^	66 ± 6	309 ± 42^a^^,^ b
Non‐HDL (mg/dL)	37 ± 7	619 ± 102^a^	49 ± 4	240 ± 47^a^^,^ b
HDL (mg/dL)	42 ± 6	65 ± 16	16 ± 2	69 ± 5
Non‐HDL:HDL ratio	1.2 ± 0.5	9.6 ± 1.2^a^	3.1 ± 0.4	3.7 ± 1.1^b^

HFD, high‐saturated‐fat diet. *N* = 6 rat dams per group. Data are shown as mean ± SEM.

a *P *≤* *0.05 compared to Anesthesia+Reg.

b *P *≤* *0.05 compared to Anesthesia+HFD.

HFD‐fed dams produced milk with greater percent lipid content and greater concentration of cholesterol, with no difference between Anesthesia+HFD and Surgery+HFD rat milk (Table 3).

**Table 3:** Maternal milk composition at postdelivery day 14

	Anesthesia+Reg	Anesthesia+HFD	Surgery+Reg	Surgery+HFD
Creamatocrit (%)	14.7 ± 0.8	20 ± 1.1^a^	14.7 ± 1.6	19.1 ± 1.8^a^
Cholesterol (mg/dL)	43 ± 4	75 ± 16^a^	40 ± 6	61 ± 11^a^

*N* = 6 rat dams per group. Data are shown as mean ± SEM.

a *P *≤* *0.05 compared to Anesthesia+Reg.

### Effect of diet and IUGR on growth and food intake

Unless otherwise specified, results described below pertain to both male and female rat offspring. Con+HFD rats weighed less than Con+Reg rats from dams on the regular diet (Table 4). Fetal IUGR+HFD rats weighed less than Con+HFD rats. While liver weight and the ratio of liver weight to body weight was decreased in IUGR+Reg, Con+HFD, and IUGR+HFD fetal rats compared to Con+Reg fetal rats, the ratio of brain weight to body weight was increased. Thus, similar to other models of asymmetric IUGR, the IUGR+HFD rats in this model have brain sparing (Simmons et al. 1992).

**Table 4:** Fetal weights obtained at the time of cesarean section and offspring body weight at PND 14

	Con+Reg	Con+HFD	IUGR+Reg	IUGR+HFD
Fetal rat				
Male				
Body weight (g)	4.3 ± 0.2	3.9 ± 0.6^a^	3.3 ± 0.4^a^	3.2 ± 0.3^a^^,^ b
Brain weight (g)	0.17 ± 0.01	0.18 ± 0.01	0.16 ± 0.01	0.16 ± 0
Brain:body weight ratio	0.04 ± 0	0.05 ± 0^a^	0.06 ± 0^a^	0.06 ± 0.1^a^^,^ b
Liver weight (g)	0.32 ± 0	0.29 ± 0.01	0.16 ± 0.02^a^	0.2 ± 0.03^a^^,^ b
Liver:body weight ratio	0.07 ± 0	0.07 ± 0	0.05 ± 0^a^	0.06 ± 0.01^a^
Placenta weight (g)	0.73 ± 0.05	0.63 ± 0.02	0.54 ± 0.04^a^	0.66 ± 0.05
Placental efficiency (g/g)	6.2 ± 0.4	6.2 ± 0.4	5.8 ± 0.5	4.9 ± 0.3^a^^,^ b
Female				
Body weight (g)	4.2 ± 0	3.6 ± 0.5^a^	3 ± 0.6	2.9 ± 0.2^a^^,^ b
Brain weight (g)	0.17 ± 0	0.16 ± 0.01	0.17 ± 0	0.15 ± 0.01
Brain:body weight ratio	0.04 ± 0	0.05 ± 0	0.06 ± 0^a^	0.05 ± 0^a^^,^ b
Liver weight (g)	0.31 ± 0.01	0.26 ± 0.02^a^	0.21 ± 0.02^a^	0.19 ± 0.03^a^^,^ b
Liver:body weight ratio	0.08 ± 0	0.07 ± 0	0.06 ± 0	0.07 ± 0
Placenta weight (g)	0.76 ± 0.07	0.67 ± 0.03	0.55 ± 0.04^a^	0.68 ± 0.04
Placental efficiency (g/g)	5.8 ± 0.5	5.5 ± 0.3	5.8 ± 0.7	4.3 ± 0.3^a^^,^ b
PND 14 rat				
Male				
Body weight (g)	36.7 ± 0.9	35.7 ± 0.6	34.2 ± 1.5^a^	33.3 ± 0.5^a^^,^ b
Female				
Body weight (g)	36.3 ± 1.1	34 ± 0.6^a^	32.1 ± 1.1^a^	31.2 ± 1^a^^,^ b

Offspring from *n* = 10 rat dams per group. Offspring body and organ weights were obtained from two male and two female offspring from each dam, averaged per sex, and the litter average per sex averaged over the 10 dams. Data are shown as mean ± SEM. HDL, high‐density lipoprotein, HFD, high‐saturated‐fat diet; IUGR, intrauterine growth restriction.

a *P *≤* *0.05 compared to Con+Reg.

b *P *≤* *0.05 compared to Con+HFD.

Placental weights were obtained as a proxy measure for surface area for maternal–fetal nutrient transfer (Gilbert et al. 2012). Placental efficiency defines the grams of rat produced per gram of placenta weight and low placental efficiency is a marker for impaired nutrient transfer. Placenta weights were significantly decreased in IUGR+Reg rats, however, no change was noted between Con+HFD and IUGR+HFD placental weights. IUGR+HFD rats had decreased placental efficiency compared to the other three groups.

At PND 14, IUGR+Reg, Con+HFD, and IUGR+HFD female rats continue to weigh less than sex‐matched Con+Reg rats, and IUGR+Reg and IUGR+HFD male rats continue to weigh less than sex‐matched Con+Reg rats (Table 4).

### Effect of diet and IUGR on cholesterol

Serum total and non‐HDL cholesterol were the same between Con+Reg, IUGR+Reg, Con+HFD, and IUGR+HFD fetal rats (Table 5). Con+HFD and IUGR+HFD rats had increased serum non‐HDL cholesterol in PND 14 rats. IUGR+HFD did not increase the ratio of non‐HDL to HDL cholesterol compared to Con+HFD rats at PND 14.

**Table 5:** Fasting serum lipid profile obtained from rat offspring at the time of cesarean section and at postnatal day 14

	Con+Reg	Con+HFD	IUGR+Reg	IUGR+HFD
Fetal rat				
Male				
Total cholesterol (mg/dL)	76 ± 3	66 ± 10	64 ± 9	60 ± 10
Non‐HDL (mg/dL)	29 ± 3	28 ± 7	27 ± 3	23 ± 1
HDL (mg/dL)	ND	ND	ND	ND
Non‐HDL:HDL ratio	NC	NC	NC	NC
Female				
Total cholesterol (mg/dL)	67 ± 8	68 ± 5	75 ± 9	58 ± 9
Non‐HDL (mg/dL)	24 ± 2	26 ± 2	27 ± 2	25 ± 4
HDL (mg/dL)	ND	ND	ND	ND
Non‐HDL:HDL ratio	NC	NC	NC	NC
PND 14 rat				
Male				
Total cholesterol (mg/dL)	158 ± 2	155 ± 4	156 ± 3	162 ± 2
Non‐HDL (mg/dL)	68 ± 2	88 ± 5^a^	73 ± 5	98 ± 2^a^
HDL (mg/dL)	67 ± 7	84 ± 20	67 ± 11	72 ± 5
Non‐HDL:HDL ratio	1.1 ± 0.1	1.7 ± 0.6	1.2 ± 0.1	1.4 ± 0.7
Female				
Total cholesterol (mg/dL)	172 ± 4	166 ± 2	166 ± 3	167 ± 2
Non‐HDL (mg/dL)	78 ± 3	103 ± 2^a^	52 ± 17	100 ± 5^a^
HDL (mg/dL)	83 ± 14	102 ± 12	37 ± 12^a^	85 ± 12
Non‐HDL:HDL ratio	1.1 ± 0.3	1.1 ± 0.1	1.5 ± 0.3	1.3 ± 0.2

Data are shown as mean ± SEM.

HDL, high‐density lipoprotein; HFD, high‐saturated‐fat diet; IUGR, Intrauterine growth restriction; ND, not detected; NC, not able to calculate. *N* = 6 per sex, diet, and surgical intervention.

a *P *≤* *0.05 compared to Con+Reg.

**Figure 2:**
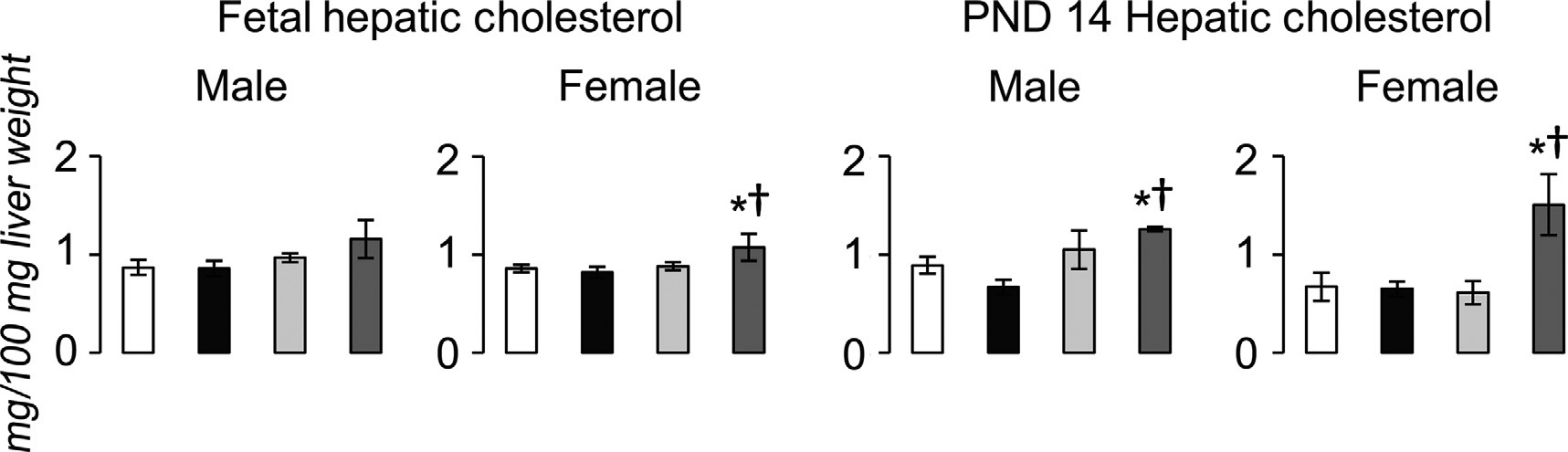
Hepatic cholesterol content in fetal and PND 14 rats. *P ≤* *0.05 compared to sex‐matched Con+Reg, †*P *≤* *0.05 compared to sex‐matched Con+HFD. Data from Con+Reg rats are shown in white bars, Con+HFD rats are shown in black bars, IUGR+Reg rats shown in light gray bars, and IUGR+HFD rats are shown in dark gray bars. *N* = 6 per sex, diet, and surgical intervention. Data are shown as mean ± SEM. HFD, high‐saturated‐fat diet; IUGR, Intrauterine growth restriction.

At birth, IUGR+Reg and Con+HFD did not alter hepatic cholesterol (Fig. 2). Compared to Con+Reg, IUGR+Reg, and Con+HFD rats, IUGR+HFD increased hepatic cholesterol in female fetal rats and in both male and female rats at PND 14.

### Effect of diet and IUGR on hepatic proteins

**Figure 3:**
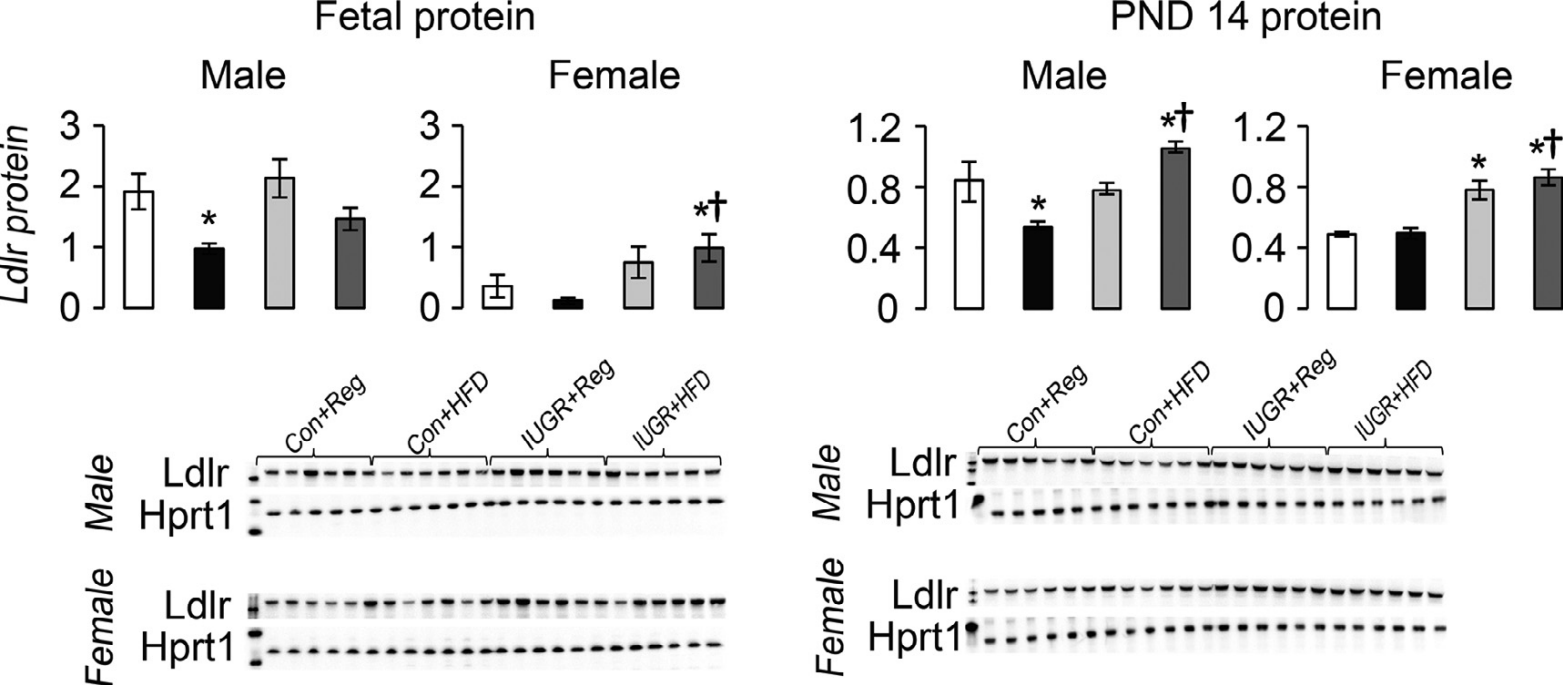
Hepatic protein levels of Ldlr relative to control protein Hprt1. *P ≤* *0.05 compared to sex‐matched Con+Reg, †*P *≤* *0.05 compared to sex‐matched Con+HFD. Data from Con+Reg rats are shown in white bars, Con+HFD rats are shown in black bars, IUGR+Reg rats shown in light gray bars, and IUGR+HFD rats are shown in dark gray bars. Western blot image is depicted below the graph, with a representative Hprt1 control blot at the bottom. *N* = 6 per sex, diet, and surgical intervention. Data are shown as mean ± SEM. HFD, high‐saturated‐fat diet; IUGR, intrauterine growth restriction.

**Figure 4:**
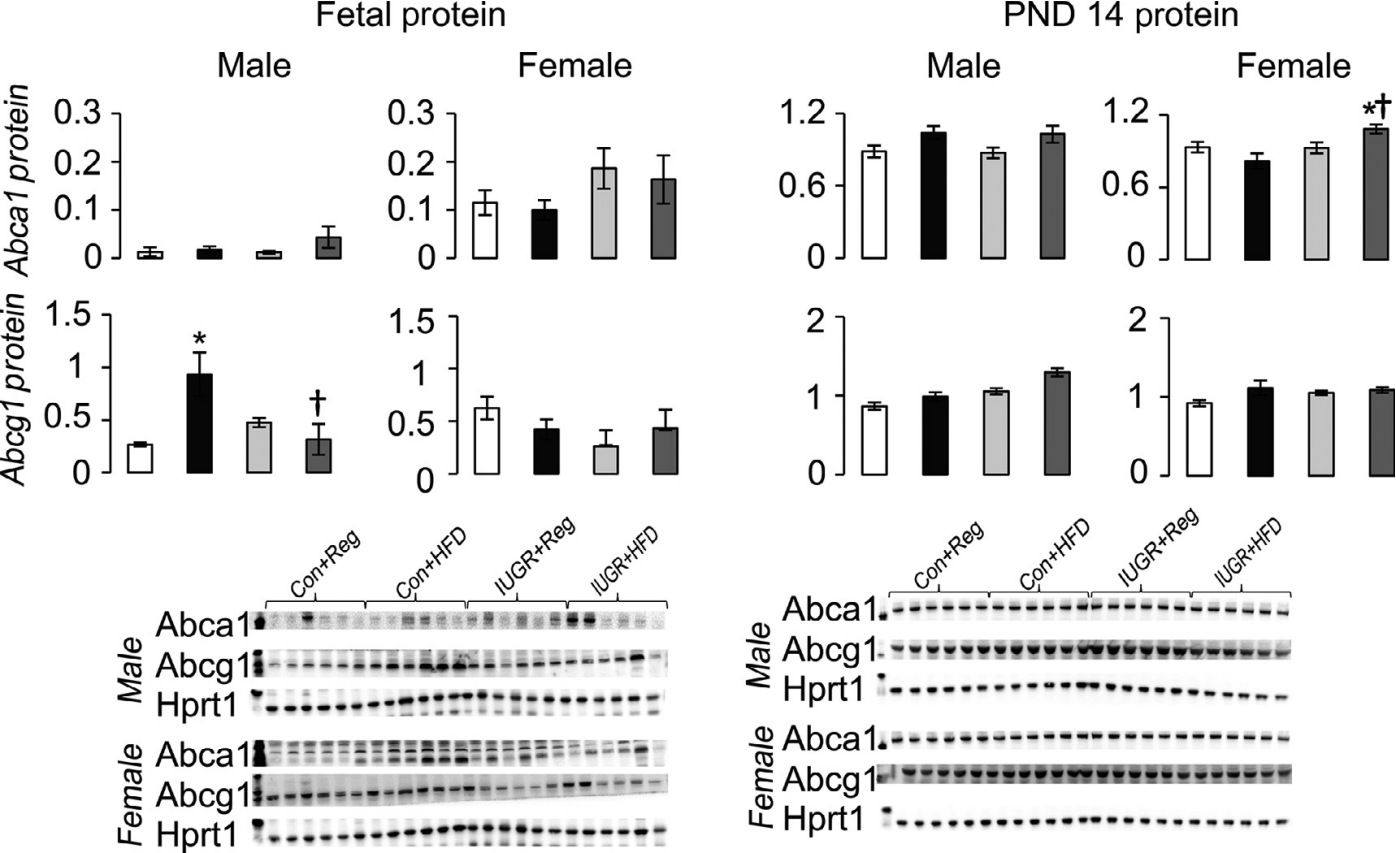
Protein levels of proteins involved in HDL uptake from the blood to the liver relative to control protein Hprt1. *P ≤* *0.05 compared to sex‐matched Con+Reg, †*P *≤* *0.05 compared to sex‐matched Con+HFD. Data from Con+Reg rats are shown in white bars, Con+HFD rats are shown in black bars, IUGR+Reg rats shown in light gray bars, and IUGR+HFD rats are shown in dark gray bars. Western blot image is depicted below the graph, with a representative Hprt1 control blot at the bottom. *N* = 6 per sex, diet, and surgical intervention. Data are shown as mean ± SEM. HDL, high‐density lipoprotein; HFD, high‐saturated‐fat diet; IUGR, intrauterine growth restriction.

**Figure 5:**
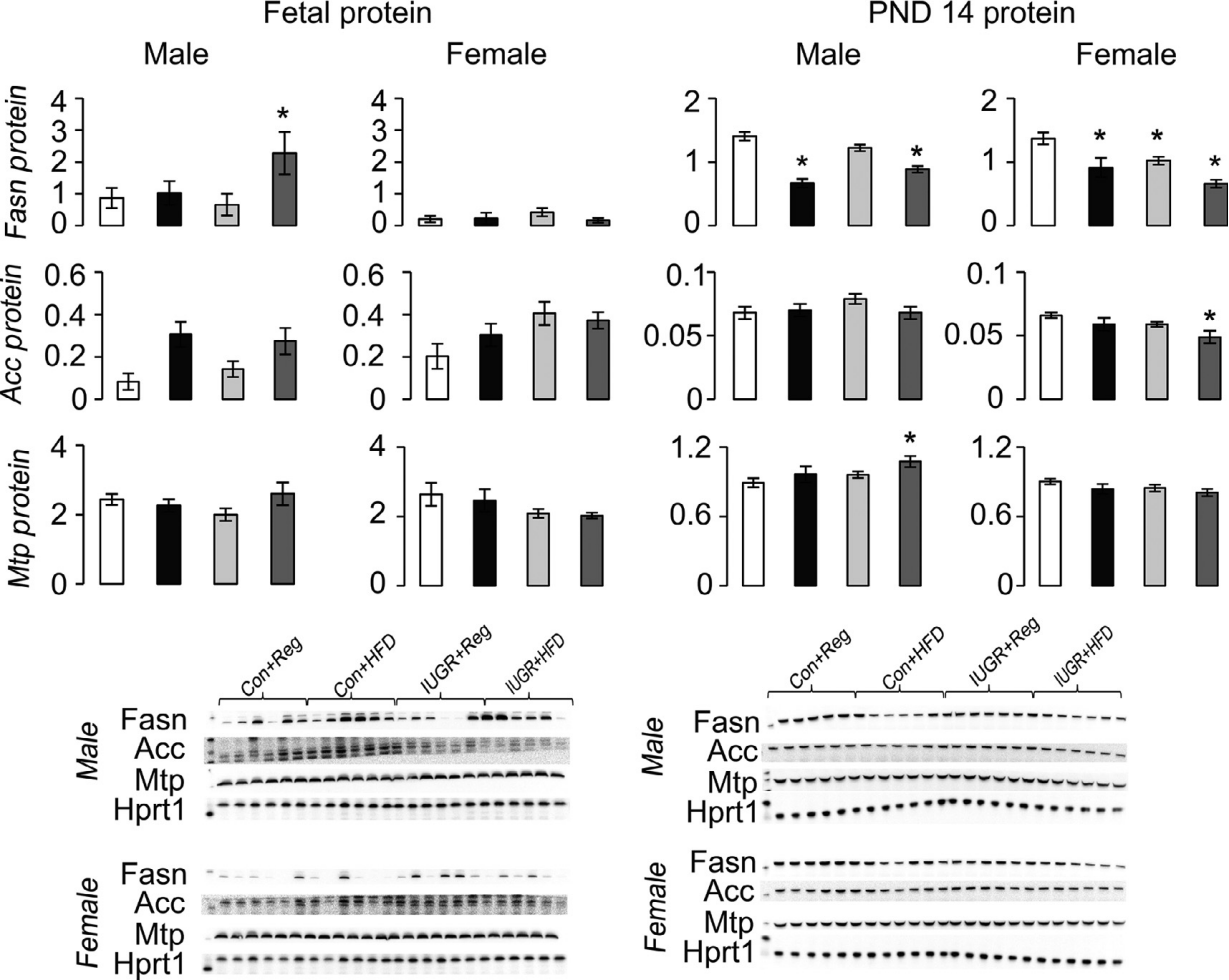
Protein levels of proteins involved in hepatic VLDL export to the blood relative to control protein Hprt1. *P ≤* *0.05 compared to sex‐matched Con+Reg, †*P *≤* *0.05 compared to sex‐matched Con+HFD. Data from Con+Reg rats are shown in white bars, Con+HFD rats are shown in black bars, IUGR+Reg rats shown in light gray bars, and IUGR+HFD rats are shown in dark gray bars. Western blot image is depicted below the graph, with a representative Hprt1 control blot at the bottom. *N* = 6 per sex, diet, and surgical intervention. Data are shown as mean ± SEM. HFD, high‐saturated‐fat diet; IUGR, intrauterine growth restriction; LDL, low‐density lipoprotein; VLDL, very low‐density lipoprotein.

**Figure 6:**
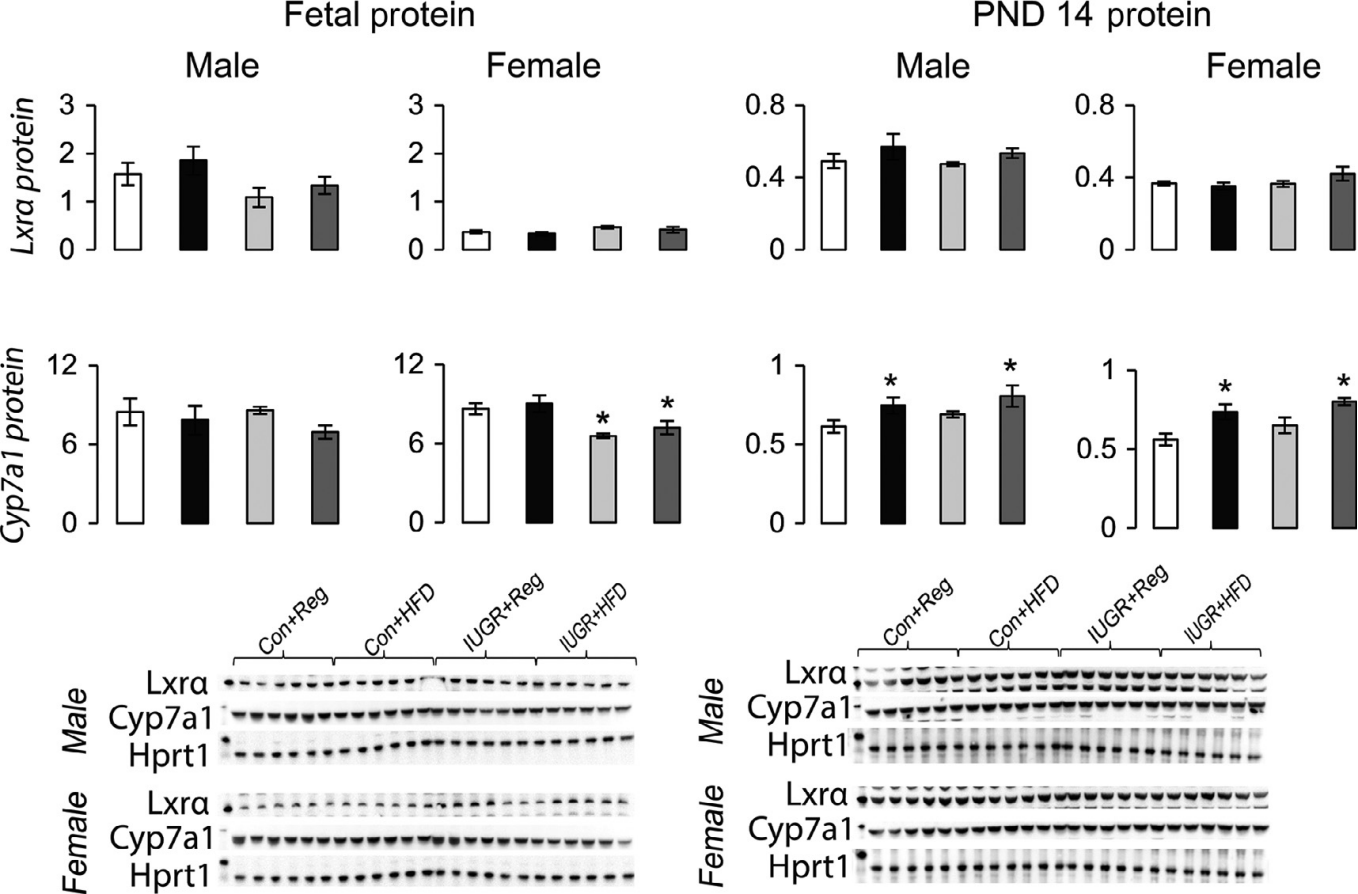
Protein levels of proteins involved in hepatic cholesterol catabolism to bile acids relative to control protein Hprt1. *P ≤* *0.05 compared to sex‐matched Con+Reg, †*P *≤* *0.05 compared to sex‐matched Con+HFD. Data from Con+Reg rats are shown in white bars, Con+HFD rats are shown in black bars, IUGR+Reg rats shown in light gray bars, and IUGR+HFD rats are shown in dark gray bars. Western blot image is depicted below the graph, with a representative Hprt1 control blot at the bottom. *N* = 6 per sex, diet, and surgical intervention. Data are shown as mean ± SEM. HFD, high‐saturated‐fat diet; IUGR, intrauterine growth restriction.

**Figure 7:**
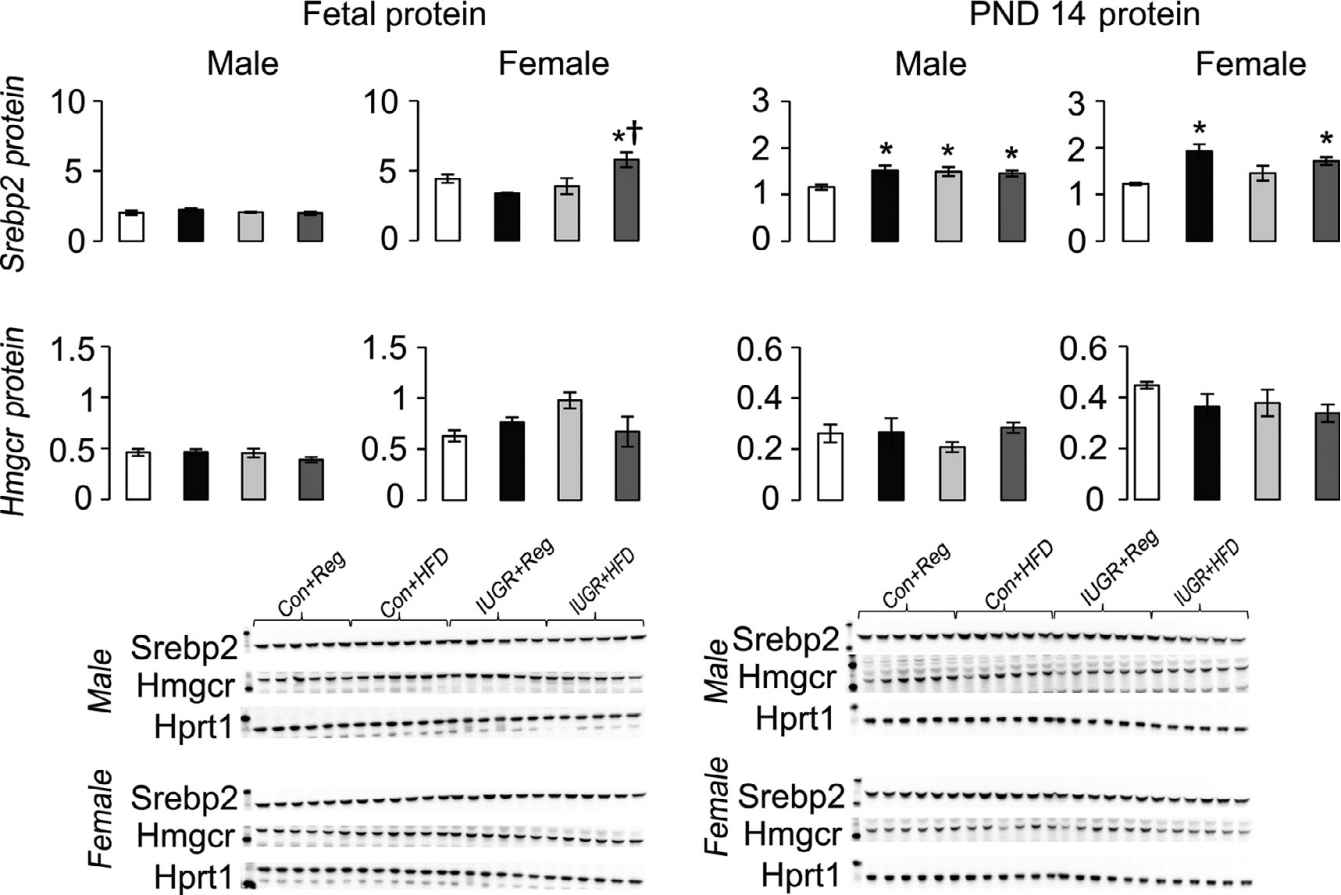
Protein levels of proteins involved in hepatic de novo cholesterol synthesis relative to control protein Hprt1. *P ≤* *0.05 compared to sex‐matched Con+Reg, †*P *≤* *0.05 compared to sex‐matched Con+HFD. Data from Con+Reg rats are shown in white bars, Con+HFD rats are shown in black bars, IUGR+Reg rats shown in light gray bars, and IUGR+HFD rats are shown in dark gray bars. Western blot image is depicted below the graph, with a representative Hprt1 control blot at the bottom. *N* = 6 per sex, diet, and surgical intervention. Data are shown as mean ± SEM. HFD, high‐saturated‐fat diet; IUGR, Intrauterine growth restriction.

Compared to Con+Reg rats, IUGR+Reg female rats had increased Ldlr protein at PND 14, but not at PND 0 (Fig. 3). IUGR+Reg male and female fetal and juvenile rats had no change in either Abca1 or Abcg1 protein levels (Fig. 4). IUGR+Reg female rats had increased Fasn protein at PND 14, but no change in Acc or Mtp protein in either sex at PND 0 (Fig. 5). IUGR+Reg rats had no change in Lxr*α*, Cyp7a1 (Fig. 6), Srebp2, or Hmgcr (Fig. 7) protein levels at either time point.

Con+HFD rat offspring had decreased Ldlr protein in male fetal and PND 14 rats compared to Con+Reg rats (Fig. 3). Con+HFD male rat offspring had increased Abcg1 protein at PND 0 but not at PND 14, with no changes in Con+HFD female Abca1 or Abcg1 protein levels (Fig. 4). Con+HFD male rats had decreased Fasn protein at PND 14, with no change in Acc or Mtp protein levels and no change in female Fasn, Acc, or Mtp protein levels at either time point (Fig. 5). Con+HFD male and female rats had increased Cyp7a1 protein at PND 14, with no change in Lxr*α* or Cyp7a1 in fetal rats (Fig. 6). Con+HFD male and female rats had increased cleaved Srebp2 protein at PND 14, but no change in Hmgcr protein at either time point (Fig. 7).

Compared to Con+Reg, Con+HFD, and IUGR+Reg rats, IUGR+HFD rats had increased Ldlr protein levels in the female fetal rats and in both sexes at PND 14 (Fig. 3). IUGR+HFD male rats had decreased Abcg1 protein compared to Con+HFD rats at birth, and female rats had increased Abca1 protein compared to both Con+Reg and Con+HFD female rats at PND 14 (Fig. 4). IUGR+HFD male fetal rats had increased Fasn protein, and at PND 14 IUGR+HFD males had decreased Fasn and Mtp protein levels (Fig. 5). IUGR+HFD female rats had decreased Fasn and Acc protein levels at PND 14. IUGR+HFD fetal female rats had decreased Cyp7a1 protein, and juvenile male and female rats had increased Cyp7a1 protein compared to Con+Reg rats (Fig. 6). IUGR+HFD fetal female rats had increased cleaved Srebp2 protein compared to both Con+Reg and Con+HFD rats, while at PND 14 IUGR+HFD male and female rats had decreased cleaved Srebp2 protein compared to Con+Reg rats (Fig. 7).

### Effect of diet and IUGR on hepatic Ldlr activity

**Figure 8:**
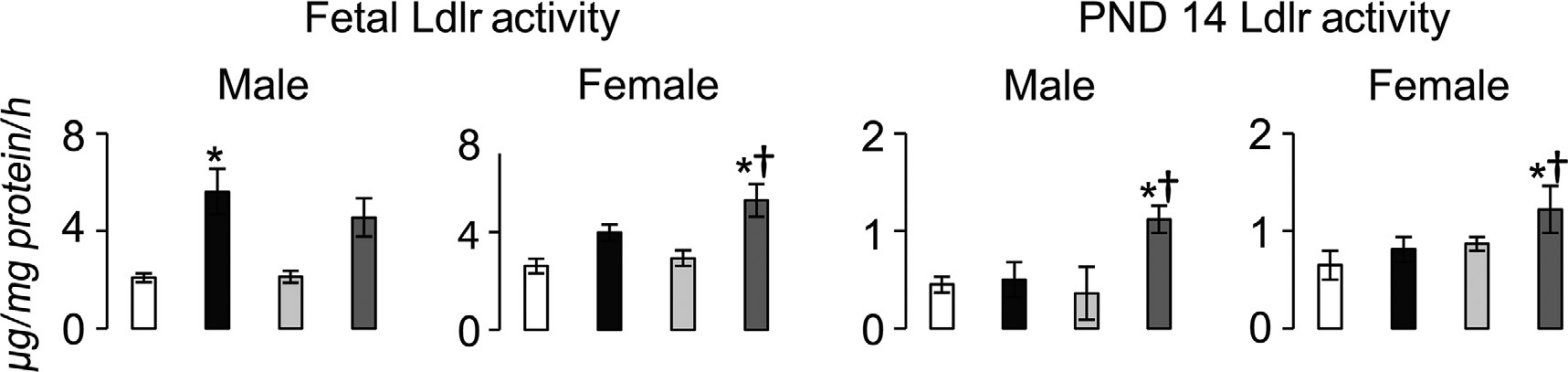
Hepatocyte LDL uptake ex vivo. *P ≤* *0.05 compared to sex‐matched Con+Reg, †*P *≤* *0.05 compared to sex‐matched Con+HFD. Data from Con+Reg rats are shown in white bars, Con+HFD rats are shown in black bars, IUGR+Reg rats shown in light gray bars, and IUGR+HFD rats are shown in dark gray bars. *N* = 6 per sex, diet, and surgical intervention. Data are shown as mean ± SEM. HFD, high‐saturated‐fat diet; IUGR, intrauterine growth restriction; LDL, low‐density lipoprotein.

Consistent with increased hepatic Ldlr protein levels in IUGR+HFD rats, hepatic Ldlr activity was increased in IUGR+HFD females at birth and in both sexes at PND 14 compared to both Con+Reg and Con+HFD rats (Fig. 8). Contrary to changes in Ldlr protein levels, Con+HFD rats had increased Ldlr activity in rats of both sexes at birth, but not at PND 14. Finally, contrary to changes in Ldlr protein levels, IUGR+Reg rats did not have changes in Ldlr activity.

## Discussion

The primary finding of this study is that only the combination of IUGR and a maternal HFD increase hepatic cholesterol accumulation in our novel rat model. Only the combination of IUGR and a maternal HFD increase Ldlr protein levels and Ldlr activity without changing protein levels of other key regulators of hepatic cholesterol metabolism. Based on Ldlr activity and protein levels of key proteins involved in the major hepatic cholesterol metabolism pathways, our findings suggest a cumulative effect of IUGR and a maternal HFD on increased cholesterol uptake from the blood to the liver via Ldlr, resulting in increased hepatic cholesterol accumulation.

The LDL receptor imports LDL cholesterol from the blood into the liver, and increased Ldlr protein levels and Ldlr activity in our study provides a potential mechanism for increased uptake of cholesterol into the liver following IUGR and a maternal HFD. Minimal literature is available on the impact of the perinatal environment on Ldlr expression. In a study of infant baboons fed either maternal baboon milk or formula, maternal baboon milk contained higher saturated fat and cholesterol compared to formula (Mott et al. 1993). Hepatic Ldlr mRNA and hepatic cholesterol were increased in maternal milk‐fed versus formula‐fed baboon livers with no change in serum cholesterol levels (Mott et al. 1993). The authors speculated that the perinatal environment influences the regulation of the Ldlr gene differently than in adults, when consumption of a high‐fat and high‐cholesterol diet decreases Ldlr expression (Sorci‐Thomas et al. 1989; Mehta et al. 1991). Our study also demonstrates the importance of the perinatal environment on Ldlr expression, though to our knowledge is the first to demonstrate an impact of the fetal environment on Ldlr protein levels. Despite the same fat and cholesterol content in maternal HFD rat milk, IUGR+HFD increased hepatic cholesterol accumulation, Ldlr protein levels, and Ldlr activity in both male and female rats at PND 14 compared to Con+HFD offspring. Increased Ldlr protein in IUGR+HFD rats suggests a role for programmed expression of Ldlr in fetal life. Serum cholesterol was unchanged by IUGR+HFD in fetal and PND 14 rats; increased import of LDL cholesterol to the liver may contribute to stable serum cholesterol levels and increased hepatic cholesterol accumulation in the offspring.

Of the key proteins representing the five major pathways through which the liver regulates cholesterol, IUGR+HFD only increased Ldlr protein levels. In our previous study of IUGR combined with a HFD in adulthood, we showed increased serum and hepatic cholesterol and decreased Cyp7a1 protein levels, with no impact on protein levels of key regulators of LDL import to the liver, HDL export to the blood, VLDL export to the blood, or de novo cholesterol synthesis (Zinkhan et al. 2014). While our current findings also demonstrate minimal impact of IUGR+HFD on levels of key proteins involved in HDL and VLDL export to the blood and de novo synthesis of cholesterol, our results do not demonstrate an impact of IUGR+HFD on Cyp7a1 protein levels at the early ages examined in this study. Thus, the perinatal environment impacts Cyp7a1 and Ldlr protein levels differently than in the adult rat. Activation of protein kinase C (Pkc) induces expression of Ldlr and decreases expression of Cyp7a1 in a time‐ and concentration‐dependent manner, providing a potential mechanistic link between our previous study and our current results (Kumar et al. 1997; Gupta et al. 2001). Increased Ldlr protein levels and stable serum cholesterol levels may be a result of fetal adaptation to in utero exposure to both a maternal HFD and IUGR. Resultant prolonged cholesterol accumulation in the liver secondary to perinatal insults may program metabolic morbidity later in life.

An adverse maternal environment impairs fetal growth. In our study, a maternal HFD increased serum cholesterol in the dams compared to regular diet‐fed dams, and maternal hypercholesterolemia is a known cause of fetal growth restriction (Montoudis et al. 1999; Bhasin et al. 2009). The combination of Surgery+HFD decreased cholesterol levels compared to Anesthesia+HFD, which may be secondary to decreased intestinal motility and nutrient absorption after surgery. Our study of maternal HFD intake and hypercholesterolemia produced a mild growth restriction of approximately 4–5%. Surgically induced IUGR in addition to maternal HFD intake produced smaller IUGR offspring compared to HFD alone, inducing an additional effect of IUGR combined with maternal HFD intake on offspring body weight at birth. Maternal HFD intake predisposes to maternal obesity and can lead to growth restriction in the fetus (Rajasingam et al. 2009). Other animal models of maternal hypercholesterolemia or HFD intake also produce IUGR offspring (Bhasin et al. 2009; Luzzo et al. 2012). The mechanism through which maternal hypercholesterolemia or a HFD lead to IUGR is unknown, though decreased nutrient delivery to the fetus has been proposed as a potential mechanism (Montoudis et al. 1999; Bhasin et al. 2009).

An important consideration in evaluation of elevated cholesterol levels in the IUGR+HFD rat offspring includes the amount of fat and cholesterol in maternal milk. High maternal dietary fat intake increases the fat content in maternal milk (Vuori et al. 1982). Furthermore, the type of fat in the maternal diet influences the fatty acid composition of maternal milk (Boris et al. 2004). In our study, while Anesthesia+HFD and Surgery+HFD dams had increased fat and cholesterol in dam milk compared to Anesthesia+Reg, there was no difference between Anesthesia+HFD and Surgery+HFD milk. Our findings suggest that offspring consumption of dam milk did not contribute to increased hepatic cholesterol levels in rats at PND 14 and emphasize the impact of the fetal environment on cholesterol levels.

Regulation of cholesterol metabolism is sex specific. IUGR induces sex‐specific increases in cholesterol levels in humans and rodents (Forsdahl 1978; Sohi et al. 2011). In humans, IUGR men had an increased ratio of LDL cholesterol to HDL cholesterol compared to IUGR women (The Hertfordshire Cohort Study, 2006). Conversely, when fed a normal‐fat diet, Ldlr knock‐out female mice had higher cholesterol compared to male Ldlr knock‐out mice (Hatch et al. 2012). In our previous study, female IUGR rats fed a HFD in adulthood had higher serum cholesterol levels than male IUGR rats fed the same diet (Zinkhan et al. 2014). Our current study similarly found sex‐specific differences in cholesterol metabolism, as female IUGR+HFD rats had increased hepatic cholesterol accumulation, Ldlr protein levels, and Ldlr activity earlier in life compared to male IUGR+HFD rats. Potential sex differences in the regulation of Ldlr protein levels in utero may result in increased hepatic cholesterol in female IUGR+HFD rats earlier in life than in male IUGR+HFD rats. Sex‐specific increases in Ldlr protein may result from increased Ldlr mRNA expression and increased accessibility of transcription machinery to the Ldlr gene. While IUGR+HFD predisposes to increased cholesterol accumulation in both sexes, earlier onset of cholesterol accumulation and therefore prolonged exposure to high cholesterol in female IUGR+HFD rats may increase the severity of long‐term metabolic complications.

A limitation of this study is that the protein and carbohydrate content of the HFD is lower than the regular diet. The protein content in the HFD was chosen to mimic protein consumption in the United States today (Wright et al. 2003) and is sufficient for normal rodent growth (FAO, WHO, 1973). Furthermore, this protein content is significantly higher than protein content used in low‐protein dietary studies (Boujendar et al. 2002). Further, unlike in humans, the HFD‐fed rats in this study consumed the same total kilocalories per day as regular diet‐fed rats. Self‐regulation of food intake was beneficial to this study as caloric intake was similar between the HFD‐fed rats and regular diet‐fed rats negating the impact of caloric intake on our results. Finally, our control group consisted of an anesthesia control and not a sham surgery control due to growth restriction induced by sham surgery (Ogata et al. 1986). Because sham surgery induces growth restriction, anesthesia alone has been used as a control in this model since 2008 (Baserga et al. 2009; Fu et al. 2009; Joss‐Moore et al. 2010, 2011a,b; Caprau et al. 2012; Fung et al. 2012; Zinkhan et al. 2014). While inclusion of a sham surgery group may be informative, we anticipate the results would be midway between the anesthesia‐alone control group and the bilateral uterine artery ligation group, and thus may not enhance our understanding of how IUGR impacts cholesterol metabolism.

In summary, only the combination of fetal IUGR and maternal HFD increase hepatic cholesterol accumulation, Ldlr protein levels, and Ldlr activity in fetal and PND 14 rats. Increased hepatic cholesterol accumulation occurs in IUGR+HFD rats without a change in dietary intake compared to Con+HFD rats. An adverse perinatal environment such as IUGR and a maternal HFD increases cholesterol accumulation that persists beyond the fetal period. We speculate that IUGR+HFD rats will demonstrate increased hepatic and serum cholesterol accumulation through adulthood resulting in increased atherosclerosis.

## Conflict of Interest

None declared.
